# Telenursing: The view of care professionals in selected EU countries. A pilot study

**DOI:** 10.1016/j.heliyon.2023.e16760

**Published:** 2023-05-27

**Authors:** Julio Emilio Marco-Franco, Margarida Reis-Santos, Isabel Barrachina-Martinez, Alina Jurewicz, Ramón Camaño-Puig

**Affiliations:** a Faculty of Nursing and Podiatry, Valencia University, Spain; b Centre of Economic Engineering (INECO), Unit of Investigation in Economy and Healthcare Management (CIEGS), Department of Economy and Social Sciences, Faculty of Business Administration and Management, Polytechnic University of Valencia, Spain; c Center for Health Technology and Services Research, Higher School of Nursing Porto, Portugal; d Abel Salazar Biomedical Sciences Institute - University of Porto, Portugal; e Department of Specialized Nursing, Faculty of Health Sciences, Pomeranian Medical University of Szczecin, Poland

**Keywords:** Telenursing, Nursing care, Enursing, Questionnaire, Rasch tests

## Abstract

**Background:**

With the growth of digital nursing, several studies have focused on recording patients' views on remote care, or specialised nurse staffing aspects. This is the first international survey on telenursing focused exclusively on clinical nurses that analyses the dimensions of usefulness, acceptability, and appropriateness of telenursing from the staff point of view.

**Methods:**

A previously validated structured questionnaire including demographic variables, 18 responses with a Likert-5 scale, three dichotomous questions, and one overall percentual estimation of holistic nursing care susceptible to being undertaken by telenursing, was administered (from 1 September to 30 November 2022) to 225 clinical and community nurses from three selected EU countries. Data analysis: descriptive data, classical and Rasch testing.

**Results:**

The results show adequacy of the model for measurement of the domains of usefulness, acceptability, and appropriateness of telenursing (overall Cronbach's alpha 0.945, Kaiser-Meyer-Olkin 0.952 and Bartlett's p < 0.001). Answers in favour of telenursing ranked 4 out of 5 in Likert scale, both globally and by the three domains. Rasch: reliability coefficient 0.94, Warm's main weighted likelihood estimate reliability 0.95. In the ANOVA analysis, the results for Portugal were significantly higher than those for Spain and Poland, both overall and for each of the dimensions. Respondents with bachelor's, master's and doctoral degrees score significantly higher than those with certificates or diplomas. Multiple regression did not yield additional data of interest.

**Conclusions:**

The tested model proved to be valid, but although the majority of nurses are in favour of telenursing, given the nature of the care, which is mainly face-to-face, according to the respondents, the chances of carrying out their activities by telenursing is only 35.3%. The survey provides useful information on what can be expected from the implementation of telenursing and the questionnaire proves to be a useful tool to be applied in other countries.

## 1 Introduction

There are four key drivers for the rapid expansion of information and communication technologies (ICT) in virtual care. Technological advances, particularly in health informatics [1,2] contributing to a better systematisation, quality control, and self-assessment of care [[3], [4], [5]]; a societal demand for equity of access [6] and for greater attention to be paid to the ageing population or to those living in remote areas [7]; labour shortage of nursing and midwifery personnel, which is predicted to worsen in the coming years [8], and cost-effectiveness constraints [9,10].

Although etymologically telenursing implies any kind of holistic remote care (e.g., ordinary mailing), it usually refers to care administered through some form of ICT system, from email, telephone, chat, or messaging [[11], [12], [13]] to incorporating computer-based videoconferencing, remote monitoring or diagnosis, or the use of remote widgets requiring complex technologies [14,15].

The various telenursing experiences published in different articles, and in few existing monographs to date [14,16], analyse aspects from the patient's perspective [17] or from that of a specific nursing speciality [[18], [19], [20], [21], [22], [23]], staff management, or promotion [4,24,25].

There is a lack of a comprehensive practical nursing assessment of the intention to use telenursing that covers practicing nurses both with and without experience in virtual care. Hopefully, our work will not only be useful for nursing care staff, but also for guiding the process of change towards digital care and reinforcing nurse participation in managerial decisions.

This cross-sectional survey aims to fill this gap by analysing the positioning of nurses from three selected EU countries in relation to remote care. The study of telenursing from the nurse's point of view, presented in this paper, has not been addressed (to our knowledge) in a comprehensive and global approach before [26,27].

## 2 Material and method

We present a cross-sectional survey (1 September to 30 November 2022) in which 225 practicing nurses from three selected EU countries (Spain, Portugal, and Poland) participated, to test their opinion regarding telenursing.

A previously validated structured questionnaire created using computer simulation [28] was self-administered, either on paper or online, which included demographic data, 18 responses (Q11−Q36) (three paired questions for each dimension) on a Likert-5 scale, three dichotomous questions (D1−D3) (one per dimension), plus an open-ended response with an overall percentage estimate of the holistic nursing care that telenursing could provide. Respondents included nurses and auxiliary nurses practicing in community centres, hospitals, or nursing homes, excluding staff involved in teaching, research, or management.

In addition to general demographic statistics, both overall and by country, the results of the polytomous questions (Q11-Q36) are presented in graphical format. The answers were reordered and re-sorted where necessary, as questions were disordered and in some cases inverted, to avoid leading questions [28]. Additionally, to detect inconsistent answers, the theoretical value of each of the three dichotomous variables (D1-D3) was calculated for each domain as the sum of the six (three pairs) values. Score was set as 1 for sums over 15. Inconsistency was considered to be found when there was a discrepancy of more than one value between reported categorical values and the theoretical values computed as above. Discrepancies were confirmed with *p*-values in Rasch person data.

Calculations were performed with the IBM-SPSS statistics package V26 for Classical Test Theory (CTT) [29]. For Item Response Theory (IRT), a Rasch analysis was performed with R (basic packages: MASS, eRm, Itm, TAM), obtaining the reliability ratio, and Warm's mean weighted likelihood estimates (WLE) reliability.

A one-way ANOVA analysis was performed using the sum of the polytomous variables as the dependent variable and country, academic degree, and workplace as independent variables. Finally, multiple linear regression modelled the total score with these variables.

Rasch analysis has also been performed including inlier-sensitive or information-weighted fit (infit-t), i.e., standardized as a z-score, standardized outlier-sensitive fit (outfit-t), betas, level of difficulty, level of discrimination, The item characteristic curves (ICC) and item information curves (IIC). Expected A Priori (EAP) and Warm's likelihood estimate (WLE) reliability ratios.

## 3 Results

### 3.1 Demographic statistics

The demographics (Table 1) show some differences in the average age of nurses, with Polish nurses about one decade younger than those of the other countries, giving an overall average of 39.18 ± 11.6 years. The majority (89.3%) of respondents were female. Years in this work provided a similar difference, matching the described differences in age (18.5–9.2), with an overall average of 14.6 ± 10.7 years. Hospital workers represented 84.0%. The post-Bologna generation, with at least a 4-year bachelor's degree, is beginning to predominate (54.7%). Almost 60% have a speciality, and 86.7% have no experience with telenursing. Respondents ranked telenursing as 4 out of 5 in the Likert scale, both globally and by the three domains of usefulness, acceptance, and appropriateness.

**Table 1:** Demographic data.

	Spain	Portugal	Poland	ALL
(*n*)	90	60	75	225
Age (Average, years)	43.6	41.4	32.2	39.18
(SD)	10.9	9.0	11.2	11.6
Females (%)	86.7	85.0	96.0	89.3
Years at work	16.5	18.4	9.2	14.6
(SD)	10.3	8.8	10.7	10.6
**Place of Work (%)**				
Hospital	84.4	65.0	98.7	84.0
Primary Care	12.2	35.0	1.3	14.7
Nursing homes	3.3	0.0	0.0	1.3
**Highest professional degree (%)**				
Nursing assistant certificate	*28.9*	*N.A.*	*0.0*	11.6
Nursing school diploma (3 years)	*32.2*	*N.A.*	*62.7*	33.8
Bachelor's degree in nursing (4 years)	*13.3*	*58.3*	*24.0*	28.9
Master's degree in nursing or health sciences	*21.1*	*40.0*	*8.0*	21.8
Doctorate in nursing or health sciences	*4.4*	*1.7*	*5.3*	4.0
N.A. Not applicable				
***Specialty****(Yes, %)*	*19.0*	*78.3*	65	58.8
**Telenursing experience (%)**				
None	84.4	86.7	89.3	86.7
Two years or less	12.2	11.7	4.0	9.3
Over two years	3.3	1.7	6.7	4.0
**Polytomous answers (Likert)**				
Global Median	4.0	4.0	4.0	4.0
Usefulness	4.0	4.0	3.5	4.0
Acceptance	4.0	4.0	4.0	4.0
Appropriateness	4.0	4.0	4.0	4.0
**Categorical answers (Y/N … % YES)**				
Usefulness (D1)	84.4	100.0	90.7	90.7
Acceptance (D2)	84.4	100.0	86.7	89.3
Appropriateness (D3)	70.6	85.0	58.7	70.5
**Estimation for telenursing use (%)**	33.5	36.3	34.5	34.3

Inconsistent careless answers were found in 16 cases (7.1%) when considering those cases where there were two or more discrepancies between the theoretical values of the categorical variables and the recorded data. It was confirmed by nonsignificant *p* values in Rasch person data. This data is in line with values reported previously [[30], [31], [32]].

### 3.2 Results for polytomous variables (Likert answers)

**Fig. 1:**
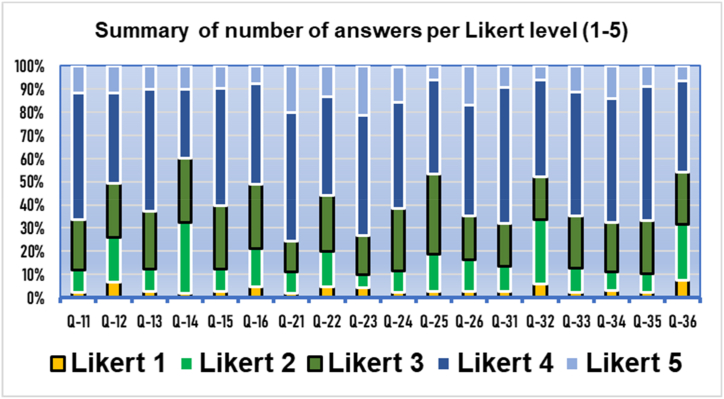
Results of the three domains by polytomous (Likert) answers. Q11−Q16 usefulness, Q21−Q26 acceptance. Q31−Q36) appropriateness. Overall results (*n* = 225).

**Fig. 2:**
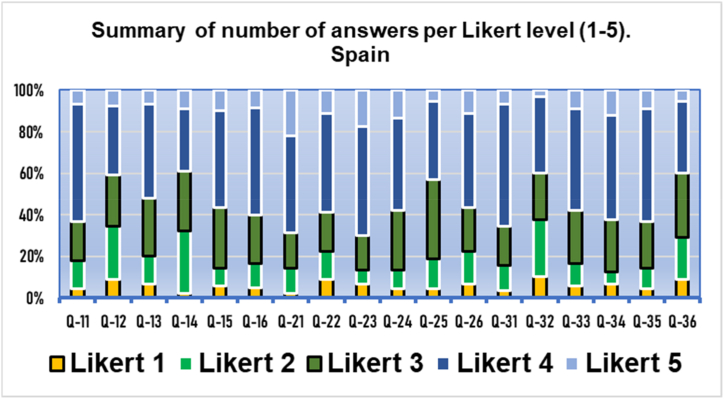
Results of the three domains by polytomous (Likert) answers. Q11−Q16 usefulness, Q21−Q26 acceptance. Q31−Q36 appropriateness. Results from Spain (*n* = 90).

**Fig. 3:**
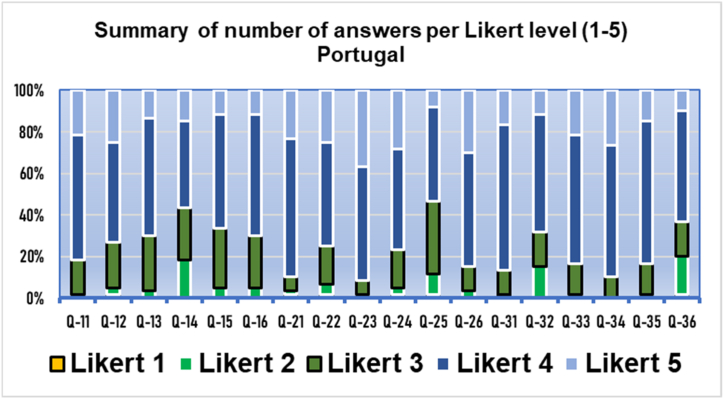
Results of the three domains by polytomous (Likert) answers. Q11−Q16 usefulness, Q21−Q26 acceptance. Q31−Q36 appropriateness. Results from Portugal (*n* = 60).

**Fig. 4:**
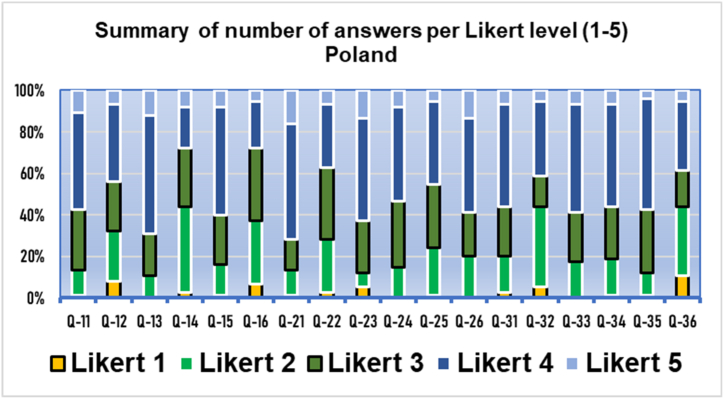
Results of the three domains by polytomous (Likert) answers. Q11−Q16 usefulness, Q21−Q26 acceptance. Q31−Q36 appropriateness. Results from Poland (*n* = 75).

These are presented in summarised graphical formats. Questions Q11−Q16 correspond to usefulness, Q21−Q26 to acceptance, and Q31−Q36 to appropriateness (Fig. 1, Fig. 2, Fig. 3, Fig. 4). Values 4–5 are predominant, indicating a positive answer.

#### 3.2.1 Overall data (n = 225)

.

#### 3.2.2 Data from Spain (n = 90)

.

#### 3.2.3 Data from Portugal (n = 60)

.

#### 3.2.4 Data from Poland (n = 75)

.

### 3.3 ANOVA results

An ANOVA analysis was performed to determine how the different parameters condition the total sum of the polytomous variables. The respondent characteristics that were significant in the ANOVA were: country, degree, and place of work. The scores obtained by respondents from Portugal are significantly higher than those from Spain and Poland, both for the total sum and for each of the dimensions. It should be noted, however, that there is no nursing assistant category in Portugal. Regarding the degree variable, groups 3, 4 and 5 (Bachelor, Master, and Doctorate) score significantly higher than groups 1 and 2 (certificate and diploma) (Table 2).

**Table 2:** Kruskal-Wallis tests.

	Sample size (n)	Average rank	Confidence intervals (95.0%)		
			Contrast	Difference	+/− Limits
**Country**					
Poland (PL)	75	93.24	PL - PT	−57.435^a^	26.992
Portugal (PT)	60	150.68	PL - SP	−11.110	24.365
Spain (SP)	90	104.35	PT - SP	46.325*	25.973
*H* = 28.6313 *p* < 0.000001	PL - PT	−57.435	*	26.992	
**Place of work**					
Hospital (H)	189	105.86	H–N	−15.1429	90.685
Nursing home (N)	3	121.00	H–P	−47.3247^a^	29.401
Primary care (P)	33	153.18	N–P	−32.1818	93.974
*H* = 14.9121 *p* < 0.001					
**Degree**					
1 Certificate	26	74.269	1–2	−20.4939	41.5155
2 Diploma	76	94.763	1–3	−58.4077^a^	42.4014
3 Bachelor	65	132.677	1–4	−56.6287^a^	44.3353
4 Master	49	130.898	1–5	−65.0641	70.6691
5 PhD	9	139.333	2–3	−37.9138^a^	30.8709
*H* = 26.3158 *p* < 0.0001			2–4	−36.1348^a^	33.4775
			2–5	−44.5702	64.4147
			3–4	34.5701	34.5701
			3–5	64.9892	64.9892
			4–5	66.2671	66.2671

a Indicates a significant difference.

### 3.4 Multiple linear regression statistical results

Multiple linear regression of the total score explains 14.7% of the variability. Age was not significant. The variables compare the Portugal group versus the rest (Spain and Poland), degree 3, 4 and 5 versus 1 and 2 and hospital as place of work versus primary care. All have been entered as two-level dummy variables and the 3 respondents from nursing homes were excluded. The mean score: increases 5.054 points on average if the respondent works in hospital versus primary care; increases 4.979 points on average if the respondent has a bachelor, master or doctoral degree versus certificate or diploma and increases 5.074 points on average if the respondent is from Portugal versus the respondents from Spain and Poland (Table 3).

**Table 3:** Multiple regression analysis summary.

Coefficients^a^								
Model		Unstandardised coefficients		Standardized Coefficients	t	Sig.	95.0% Confidence interval for B	
		B	Std. Error	Beta			Lower Bound	Upper Bound
1	(Constant)	58.128	1.183		49.148	.000	55.853	60.460
	place	5.054	2.342	.143	2.158	.032	0.824	9.578
	degree	4.979	1.878	.197	2.651	.009	1.275	8.602
	Portugal	5.074	2.195	.179	2.311	.022	0.744	9.325

a Dependent variable: Total score.

### 3.5 Classical theory results

Following our previous methodology, Table 4 summarises the non-standardised Cronbach's alpha of the polytomous variables for each database (based on covariance analyses) for each dimension. The 95% confidence intervals (CI) for Cronbach's alpha have been computed using the interclass correlation coefficients (two-way random consistency) model. Principal component analysis was performed to assess the strength of the partial correlation between the items, with Kaiser-Meyer-Olkin (KMO) and Bartlett's tests. All Cronbach's alpha excluding one question are over 0.94. Best exclusion is for question Q14 (*α* = 0.951).

**Table 4:** Principal component tests for polytomous variables by domain and overall.

Domain	Cronbach's alpha	Interclass correlation	Interval (95%CI)	KMO	Bartlett
Usefulness (D1)	0.785	0.785	0.739–0.826	0.819	<0.001
Acceptance (D2)	0.878	0.878	0.851–0.901	0.884	<0.001
Appropriateness (D3)	0.883	0.883	0.857–0.905	0.871	<0.001
Overall	0.945	0.945	0.934–0.955	0.952	<0.001

### 3.6 IRT. Rasch analyses

The results are summarised in the following table (Table 5).

**Table 5:** Summary of Item Response Theory data (Rasch test).

Variables	Index	Value	
Dichotomic	EAP reliability.	0.52	●The reliability score test to characterize the latent factor structure of the set of binary and ordered-category variables
	WLE reliability.	−0.49	
	Andersen LR test	22.32	● Andersen likelihood ratio test for the goodness of fit of the Rasch model.
	*p*	0.81	
	D1-Infit-t	−1.07	●The measurement of infit and outfit in Rasch analysis refers to the ability of the data to fit a measurement model. The infit refers to the fits of the data to the expected distribution. Infit values are often used to detect anomalous patterns in the data, such as erroneous responses or over-responses. Outfit values, on the other hand, are used to assess the predictive ability of the data. It refers to the fits of the data to the structured, default distribution.
	D2-Infit-t	−2.84	
	D3-Infit-t	−2.03	
	D1-Outfit-t	−1.12	
	D2-Outfit-t	−2.75	
	D3-Outfit-t	−3.09	●Standardised infit and outfit (-t or Z) acceptable values range −1.9 to +1.9. Over 2 data are unpredictable. Data under −2 are too predictable.
	Beta-D1 (0.95CI)	−1.39	
	Beta-D2 (0.95CI)	−0.91	
	Beta-D3 (0.95CI)	2.29	
	D1-Dffclt	−1.40	●Dffclt and Dscrm are both measures of difficulty in Rasch analyses. Both measures can be used to assess the difficulty level of a particular item, as well as the ability of individuals to respond to different difficulty levels. Dffclt is the difficulty level of a particular item on a measure, and Dscrm is the ability of an individual to discriminate between items of different difficulty levels.
	D1-Dscrmn	4.56	
	D1-P(x = 1|z = 0)	1.00	
	D2-Dffclt	−1.31	
	D2-Dscrmn	4.56	●P(x = 1|z = 0) is the probability that a person with a z-score of 0 will have an x-score of 1. This probability is determined by the person's ability level and the difficulty of the item. In this case the probabilities are around 1 in the three cases.
	D2-P(x = 1|z = 0)	1.00	
	D3-Dffclt	−0.53	
	D3-Dscrmn	4.56	
	D3-P(x = 1|z = 0)	0.92	
Polytomous	EAP reliability.	0.95	
	WLE reliability.	0.94	

In addition to the EAP, WLE reliability scores, a complete Rasch test was performed for the categorical variables, although its value considering only three categorical questions cannot be taken into account alone.

**Fig. 5:**
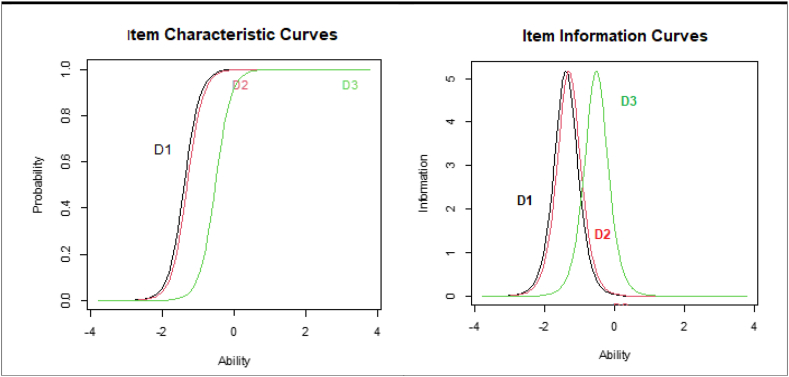
Item Characteristic Curves (ICC) and the Item Information Curves (IIC) for categorical variables.

The following graphics, included in Fig. 5, depict the Item Characteristic Curves (ICC) and the Item Information Curves (IIC) for categorical (D1−D3) variables.

The IIC and ICC curves are used to assess the internal consistency of the Rasch data. The ICC curve measures the reliability of the measurement and assesses whether the measurement instrument is reliable in measuring the desired construct. The slope of the ICC represents how much the probability of giving a positive answer increase for supporters of telenursing.

There is a left displacement of the ICC, particularly for usefulness and acceptance. This is probably due to a high degree of ‘ceiling’ effect, meaning that the items are too easy for the majority of the test takers, together with an unequal distribution of scores, resulting in a large number of test takers scoring at the same (positive) level. An average respondent has a probability over 90% in all the cases.

The IIC peak at the difficult value (highest discrimination of the item). It provides information on how much ‘information’ about the latent trait ability is given by an item. The curves are again left biased. The most likely reason for the left shift of the IIC Curves is that the items are too easy for the group of test subjects.

The real value of the Rasch analyses rely on the reliability coefficients for polytomous questions (0.94–0.95).

## 4 Discussion

Technological factors, increase in community needs, severe staff shortages and cost effectiveness will drive the growth of telenursing, which needs to include the vision of nurses and their active participation in the design and implementation of the programme as essential elements for success [33,34]. Often, organisational changes in health care start from the upper levels of management, with a top-down approach, where not infrequently the professional community is ignored. This leads to delays, if not the abandonment of the new procedure [33,34].

As reported, our questionnaire [28] has been inspired by previous experiences of healthcare staff already teleworking [[35], [36], [37], [38]]; by general questionnaires on the incorporation of technologies [39,40]; and by a previous design focusing on patients' behaviour towards telenursing (TISQ) [17].

Taking these previous experiences into account, and focusing on eliciting the essential elements [41], careful attention has been paid to avoid the frequent error of leading questions [42]. Therefore, each question is paired with an opposing question and the pairs were randomly unordered so that the questions cannot be answered routinely. This allows testing for consistency.

Following the philosophy of the TSQ questionnaire, designed specifically for telemedicine systems, it is assumed that the equipment will be used by patients and professionals once it has been installed and tested, so technical questions relating to the interface have not been included. However, some related aspects have been included, inspired by the TAM, a questionnaire not specific to health sciences but widely used in the commercial world and which includes questions related to usability factors of usefulness and ease of use [39,40].

Details on Classical Test Theory methodology (for polytomous variables) can be found in our previous work [28]. Cronbach's alpha, KMO and Bartlett's tests confirm the adequacy of the results. Overall, Cronbach's alpha is very high (0.945), with interclass correlation of 0.940. KMO 0.952 and Bartlett p < 0.001 (Table 4).

The Kaiser-Meyer-Olkin Sphericity Test (KMO) assesses the correlation between variables and determines whether the data set is adequately related to apply dimensionality reduction. This means that it measures the degree to which a set of variables is related to each other. A KMO score above 0.6 indicates that the set of variables is sufficiently related to perform a factor analysis.

Bartlett's test of sphericity (Bartlett's method of covariance and varimax rotation) tests whether the correlation matrix is an identity matrix. It is used to determine whether variables in a data set were correlated. It tests the null hypothesis that all of the variables are uncorrelated against an alternative hypothesis of at least one pair of variables being correlated. If *p* is not significant, principal component analysis is not appropriate.

As expected with IRT analysis when considering a construct with only three categorical variables, the EAP reliability is 0.52, indicating an insufficient fit of the Rasch model when only dichotomous variables are included; this is confirmed by WLE of −0.49. Therefore, polytomous analysis is imperative, or else a new design of the test with no less than six categorical questions per domain should be included. In other words, the short version of using only dichotomous questions is not appropriate. The dichotomous variables provide usefulness, basically when tested against the theoretical expected values D1T−D3T obtained from Likert responses for testing careless (inconsistent) answers.

The real utility results of the IRT analysis to be evaluated for polytomous variables is the EAP reliability of 0.95, indicating that the rating scale fits the underlying Rasch model very well. It has been described that there are no major differences between different likelihood estimates (LE) approaches [43]. We have used the Warm's likelihood estimate (WLE), again providing a very good result (0.94).

Although telenursing is a promising technique and will undoubtedly be implemented progressively, the results obtained show that, according to the average of the responses obtained, the degree of application to nursing care will be limited due to the fundamentally face-to-face component of the professional activity.

This study has some limitations. The survey has been carried out in three EU countries. It remains to be analyzed in the future whether the results obtained can be replicated in other contexts and health systems, particularly in developing countries and in non-public health systems.

## 5 Conclusions

The results are not unexpected. Most nursing care needs face to face activities, but even so, there is a substantial part of the activity that practicing nurses consider may be covered by e-nursing.

It is not necessary for a telenursing programme to start with a complex computer system that allows for comprehensive patient care, management, quality control and includes a decision tree. Dedicating a few hours of professional activity to telephone (and/or video telephonic) care through an appropriately designed programme in collaboration with nursing, could be a perfectly reasonable start.

This questionnaire meets the expected objective as a starting tool to guide the process of change towards digital care and reinforcing nurse and patient participation in managerial decisions in three selected countries and could be extended to analyse the issue in the rest of EU countries.

As for the future, unless complex Rasch developments for polytomous variables are to be used, consideration could be given to transforming these questions into a series of categorical variables to facilitate Rasch analysis.

This pilot study proves that this questionnaire is robust and allows separate analysis of the three domains (usefulness, acceptability, and appropriateness). Categorial data should not be used alone, but in conjunction with polytomous data. Results show a favourable opinion from nurses about telenursing, although they consider that appropriateness for providing holistic nursing care is limited (34.3%).

## 6 Summary table

What was already known on the topic?

● The various telehealth experiences analyse aspects from the patient's or from the doctor's perspective. In telenursing, only aspects of a specific nursing speciality or aspects of management and teaching have been analyzed**.**
● The implementation of a telenursing programme requires knowledge of the acceptance, usefulness, and appropriateness of the programme by the personnel who will use it. This information was missing.
What did this study add to our knowledge?
● The development and validation of a questionnaire to analyse telenursing.
● Acceptance of telenursing is generally high among nursing staff, although it is not the same in all countries and is mostly related to the nurse's academic level, regardless of age. Even considering all aspects (holistic approach), telenursing, according to the respondents, is only applicable in about one third of the nursing procedures.

## Author contribution statement

Julio Emilio Marco-Franco: Conceived and designed the experiments; Performed the experiments; Analyzed and interpreted the data; Wrote the paper.

Ramón Camaño-Puig: Conceived and designed the experiments; Performed the experiments; Analyzed and interpreted the data; Contributed reagents, materials, analysis tools or data; Wrote the paper.

Margarida Reis-Santos: Performed the experiments; Analyzed and interpreted the data; Contributed reagents, materials, analysis tools or data; Wrote the paper.

Isabel Barrachina-Martinez: Performed the experiments; Analyzed and interpreted the data.

Alina Jurewicz: Performed the experiments; Analyzed and interpreted the data; Contributed reagents, materials, analysis tools or data.

## Data availability statement

Data will be made available on request.

## Declaration of competing interest

The authors declare no conflict of interest.
