# Supplementary material for: Telenursing: The view of care professionals in selected EU countries. A pilot study
Source: Heliyon. 2023 May 27;9(6):e16760. doi: 10.1016/j.heliyon.2023.e16760 (PMC10258424; doi:10.1016/j.heliyon.2023.e16760)

**QUESTIONNAIRE**

**Instructions on how to complete the multiple-choice questionnaire**

*This is a study carried out by the universities of Valencia (UV, and UPV), Murcia, Oporto and to gather the nursing point of view on telenursing. The person in charge of the study is Dr Julio E. Marco Franco (juemar@alumni.uv.es). Any questions you may have can be sent to this e-mail address.*

1. This is a voluntary and unpaid questionnaire which is expected to be useful to the nursing community.

2. This survey is focused on clinical nurses only. Nurses involved in management, executive functions, governmental or public health activities, research and teaching should not complete the questionnaire.

3. If you have not had previous experience in tele-nursing, try to give your opinion in the case of joining a team with such a system, based on your previous experience in telephone patient care and use of digital media (smartphones, email, social networks, music consumption in the cloud, etc.)

4. The results are completely anonymous. There are no good or bad answers. Any answer is valid and appropriate. Please tick the answer that most closely matches your opinion. Only one answer per numbered item, please.

5. If you provide the answers in paper format, please mark a single circle with an X to indicate the answer that you think best represents your opinion for each question. Please remember, only one answer out of the five possibilities.


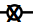


6. If you make a mistake, mark the wrong answer with a square and mark the correct answer as indicated


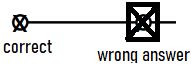


7. If you answer online, the system will allow you to change the answer. There is no need to answer all the questions at a time. Please take a few days if needed to answer, but please try to answer all questions. Submit the format when ready.

8. Remember that the system will not request or store any personal information; if you log in again from a different computer or smartphone your previous information will be lost.

8. The study follows the Principles of the Declaration of Helsinki (World Medical Association 2013). The data is recorded anonymously without any identification reference. No information reported in this document can be linked to any individual. The study has been reviewed by the Ethics Commission of the University of Valencia.

9. Thank you very much for your collaboration!

**General demographic data (anonymous).**

Please do not include any information that could identify yourself.

A. Birth year (xxxx) (year only)

|  |  |  |  |
| --- | --- | --- | --- |

B. Gender

| Male.................................................................... |  |
| --- | --- |
| Female................................................................ |  |
| Other................................................................... |  |

C. Number of years working as nurse (xx)

|  |  |
| --- | --- |

D. Country. Please mark X.

| Spain.................................................................... |  |
| --- | --- |
| Portugal................................................................ |  |
| Poland.................................................................. |  |

E. Workplace (If unemployed, please indicate the area in which you have been employed the longest). Please Mark X

| Primary and community care nurse..................... |  |
| --- | --- |
| Hospital Nurse..................................................... |  |
| Nursing home nurse............................................ |  |

F. (Highest) academic degree. If you have several degrees, please mark X only the highest one.

| Nursing assistant certificate................................. |  |
| --- | --- |
| Nursing school diploma (3 years)........................ |  |
| Bachelor's degree in nursing (4 years)................ |  |
| Master's degree in nursing or health sciences.... |  |
| Doctorate in nursing or health sciences.............. |  |

**Domain of professional practice and quality**

1. Telenursing is a good way of health care delivery (TMPQ)


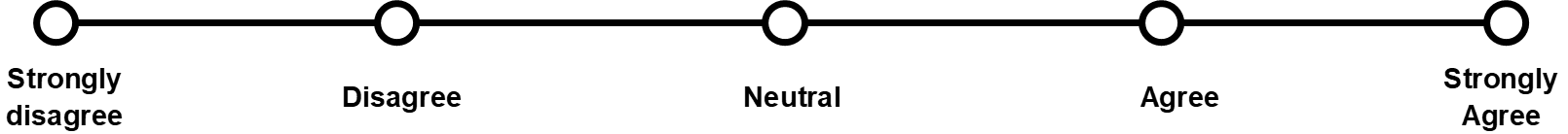


2. Telenursing does not scare me at all (TAM3-CANX)


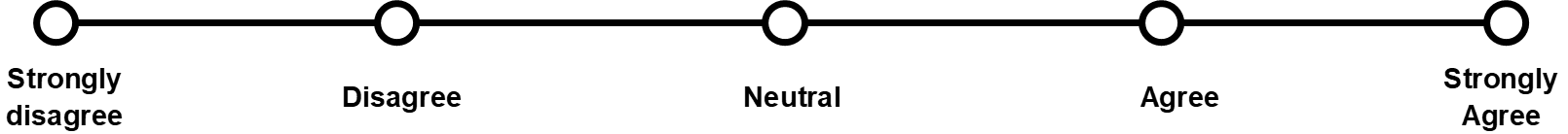


3. Telenursing allows me to listen the patient attentively (TISQ)


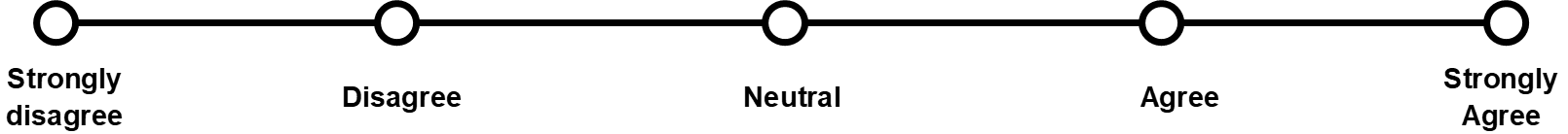


4. Telenursing allows me to provide quality and compassionate care (TISQ)


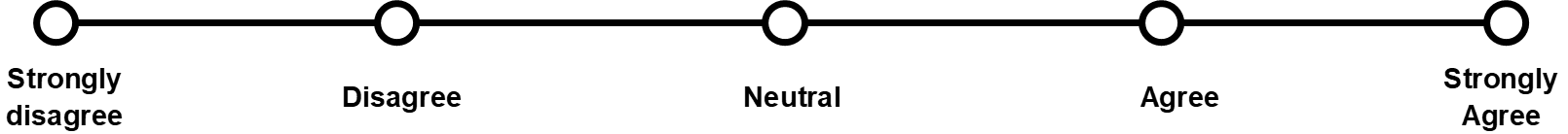


5. I can offer friendly behaviour and empathy to the patient with telenursing (TISQ)


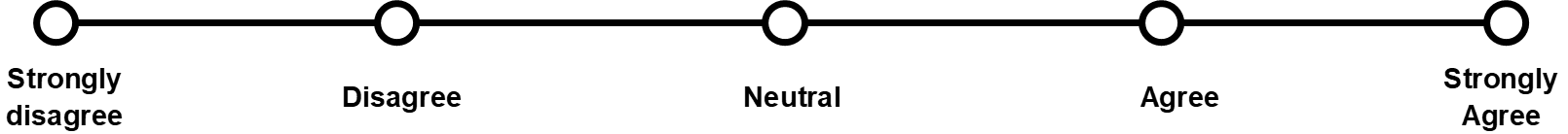


6. Telenursing allows for a detailed and thorough work as nurse (TISQ).


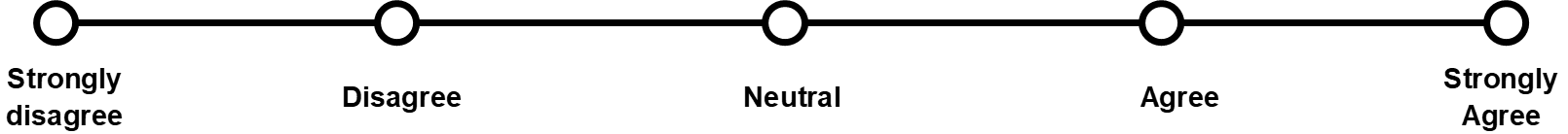


7. I feel competent to deal with the health problem using telenursing (TISQ)
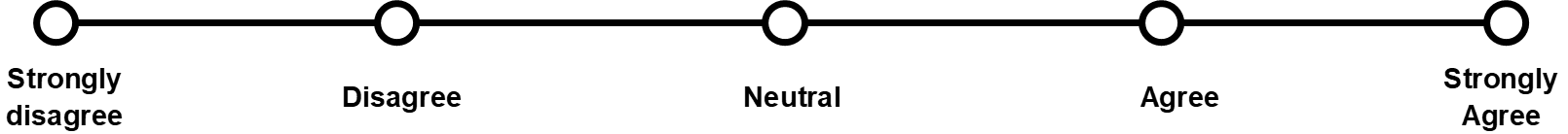


8. Telenursing helps me to better manage the health and needs of my patients (ATA)
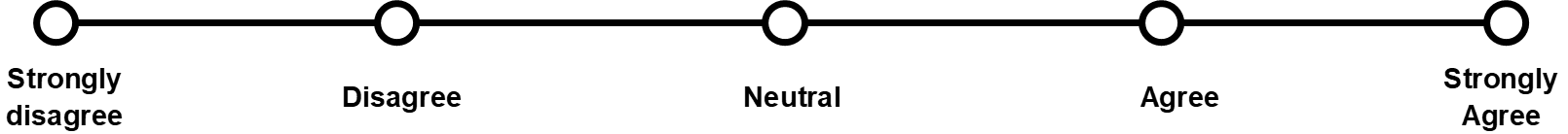


9. Telenursing gives me a good understanding of the patient's problem (TMPQ)


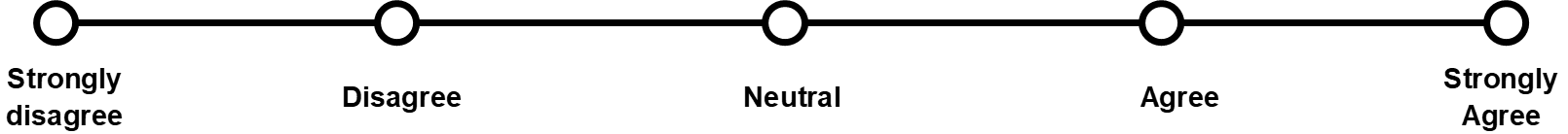


10. Overall, I am satisfied with incorporating telenursing to nursing practice (TISQ)


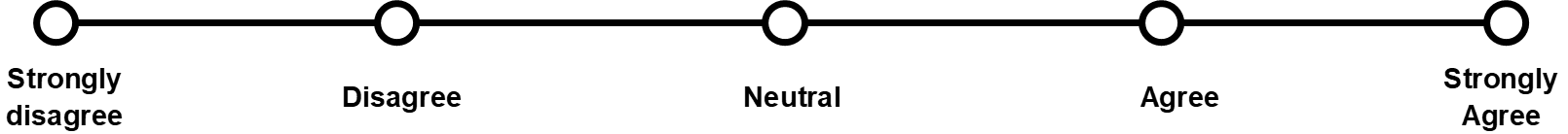


**Domain of management/productivity/usefulness**

11. I think I will use telenursing very frequently (SUS)


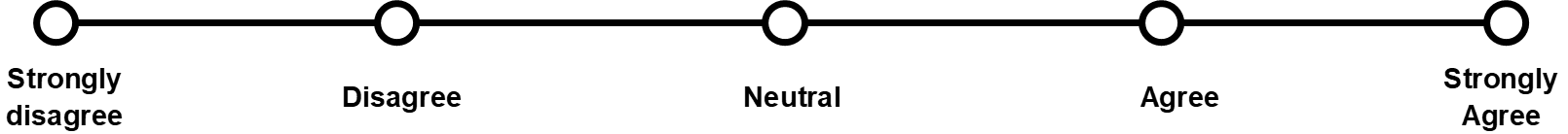


12. Telenursing increases my productivity (TAM3-PU2)


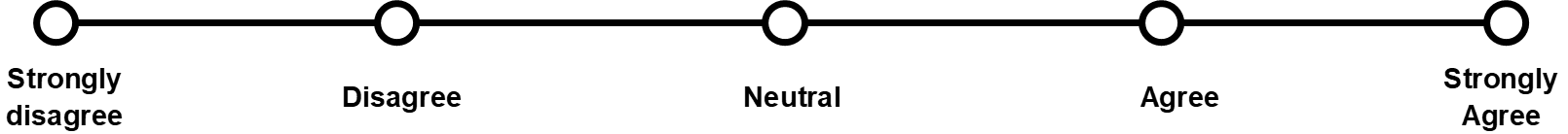


13. Telecare does not violate patients' privacy (TMPQ)


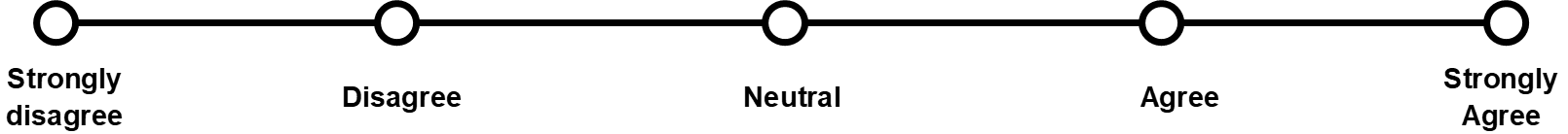


14. Telenursing optimises contact with the patient (TMPQ)


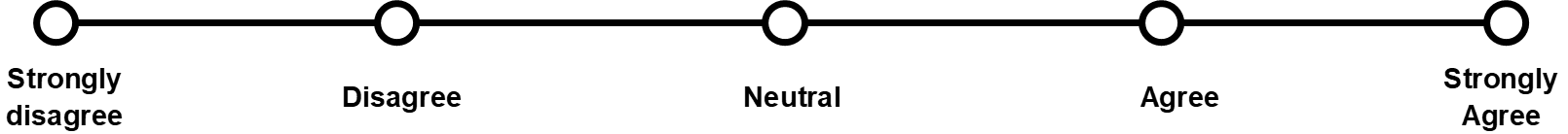


15. Telenursing improves my job performance (TAM3-PU1)


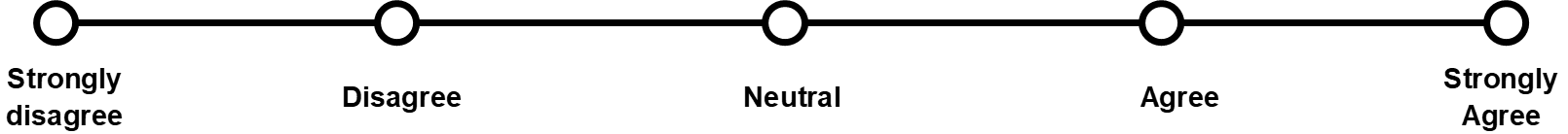


16. Telenursing does not increase professional liability and legal risks (NEW)


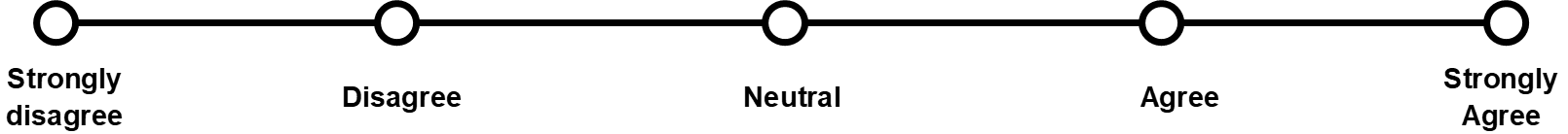


17. Telenursing enhances my effectiveness on the job (TAM3-PU3)


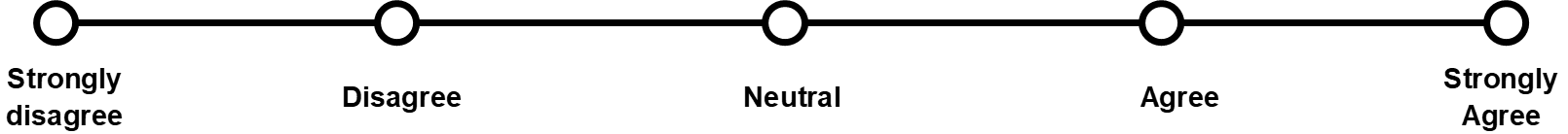


18. Telenursing saves time (TMPQ).


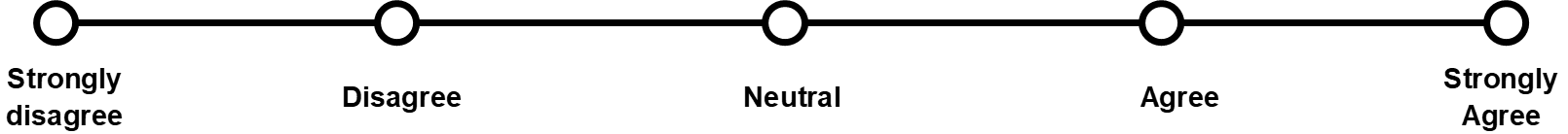


19. Telenursing enables good management of patients' needs (ATA)


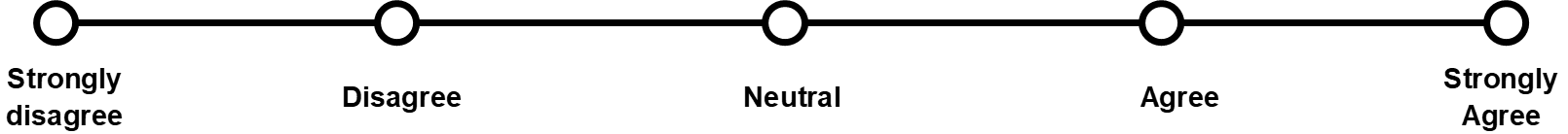


20. Overall, I find telenursing system useful in my job (TAM3-PU4)


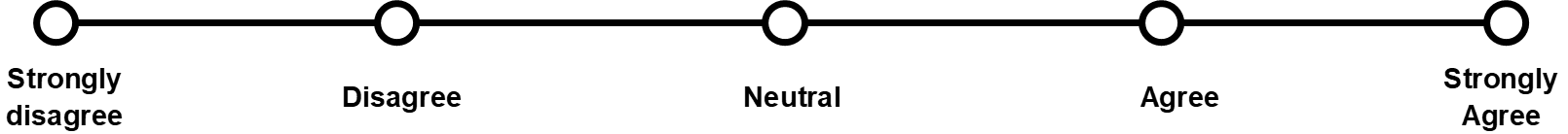


**Aspirations and career accomplishment domain**

21. Voluntary telenursing is a good professional alternative in case of maternity, parental leave, early retirement or reduced physical capacity (NEW)


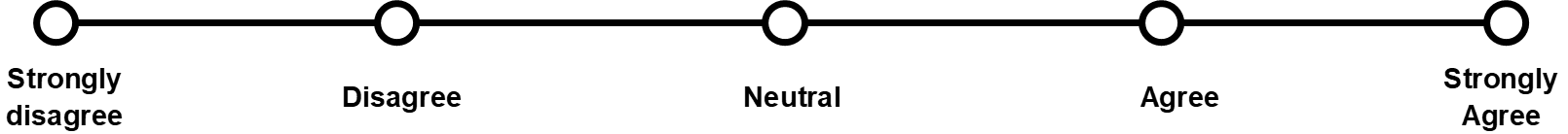


22 It will be alright if the organisation requires me to use telenursing (TAM3-SN)


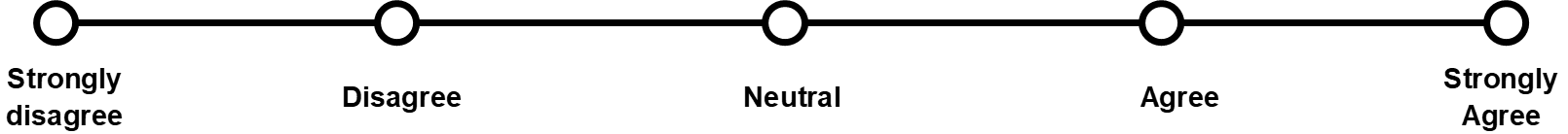


23. Training in telenursing will give me professional competitive advantages in the future (NEW)


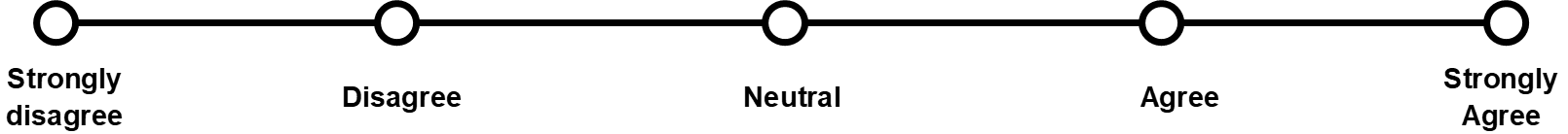


24. I believe that telenursing will be economically positive for me in my future.


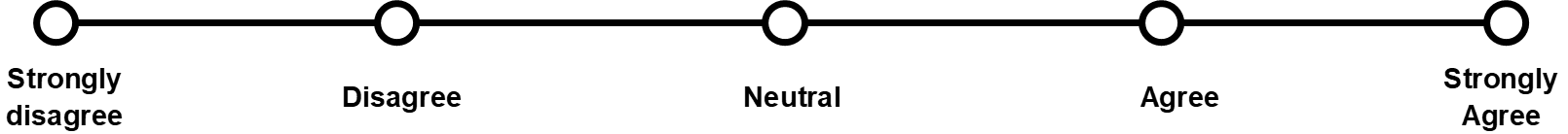


25. I think telenursing systems are important in nursing work. (TAM3-REL1)


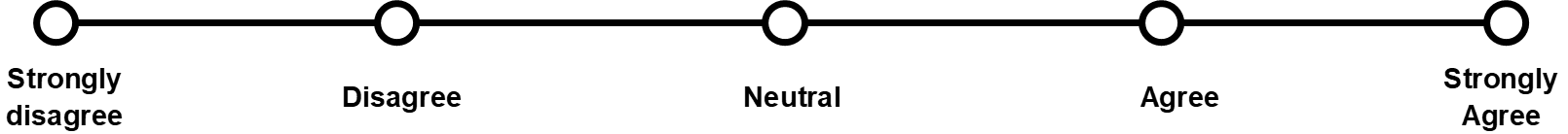


26. Being part of a virtual team providing telehealth enhances my professional development (NEW)


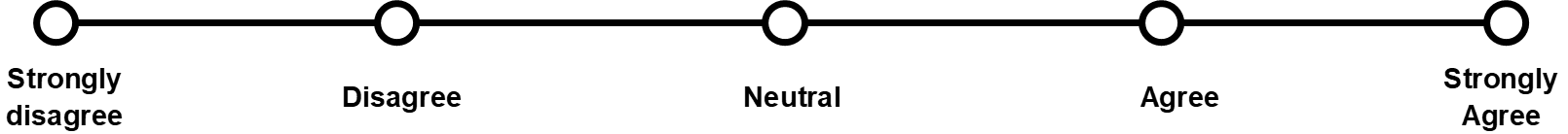


27. Telenursing allows me to optimise and better use my strengths or skills contributing to my professional satisfaction (NEW)


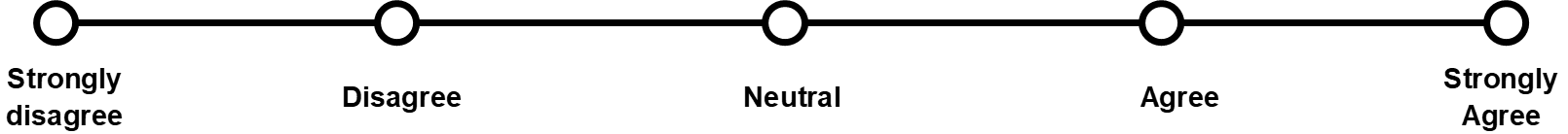


28. The organisational flexibility and my own time management make telenursing a positive contribution to my continuing education (NEW).


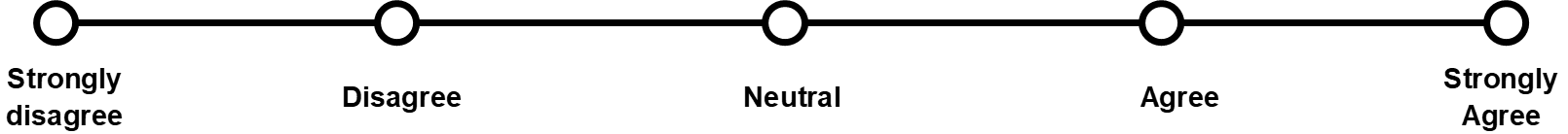


29. Being able to reach people at a distance or with access difficulties gives me professional satisfaction (NEW)


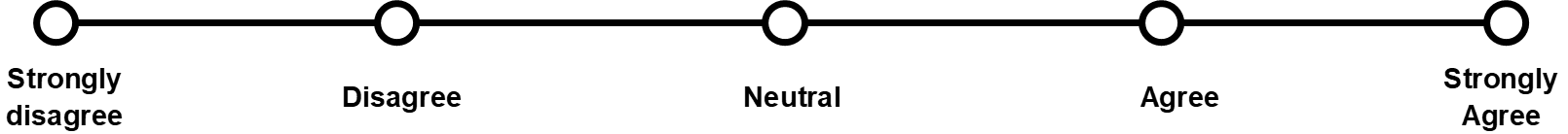


30. Overall, I consider telenursing to be positive for my professional development.


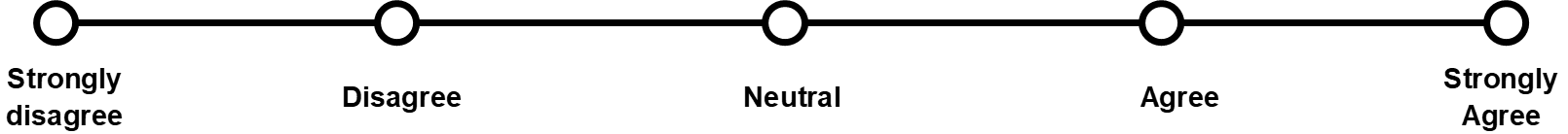


**Technical domain**

31. I find using telenursing enjoyable (TAM3-ENJ)


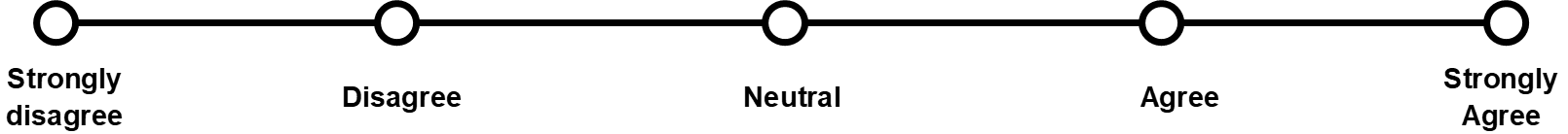


32. I imagine that most nurses would learn to use this system very quickly (SUS)


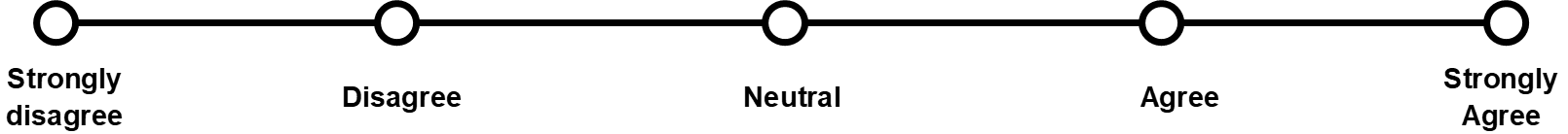


33. I find telenursing system is easy to use (TAM3-PEOU3)


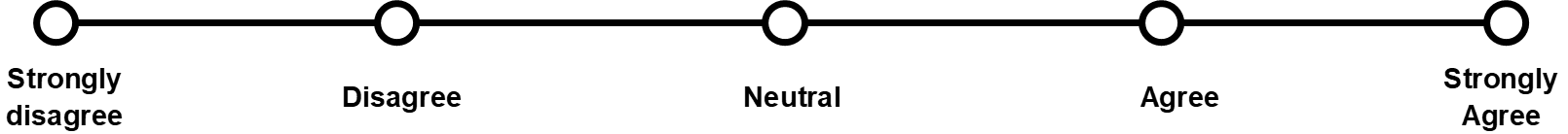


34. I find easy to get the telenursing system to do what I want it to do (TAM3-PEOU4)


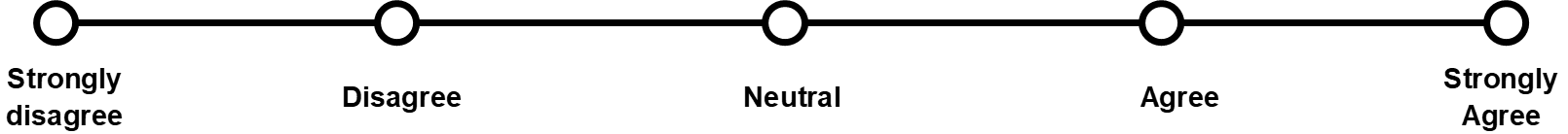


35. It is easy for me to remember how to perform tasks using telenursing (TAM)


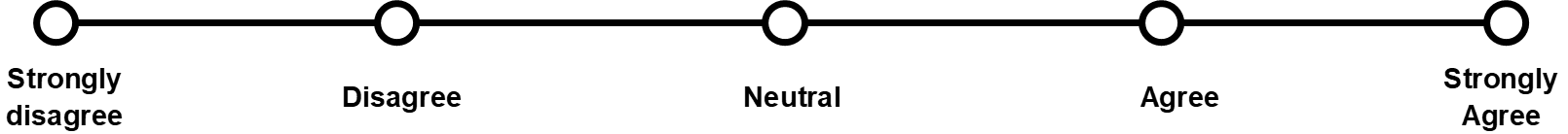


36. Use of a telenursing system does not make me feel uncomfortable (TAM3-CANX)


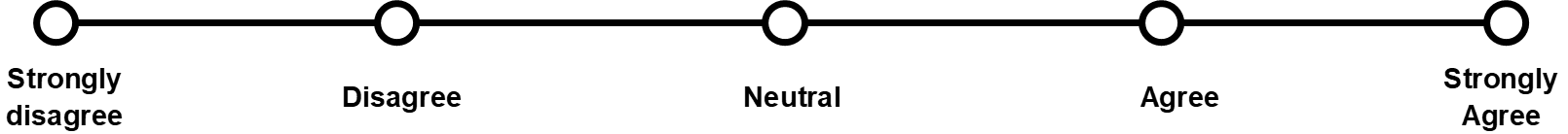


37. I find telenursing does not require a lot of my mental effort (TAM3-PEOU2)


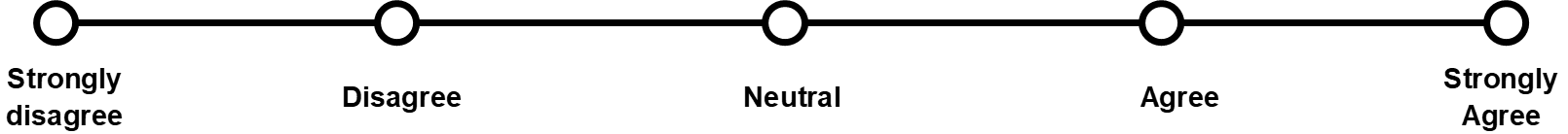


38. The built-in help facility aid software to guide nursing care is positive (TAM3-CSE)


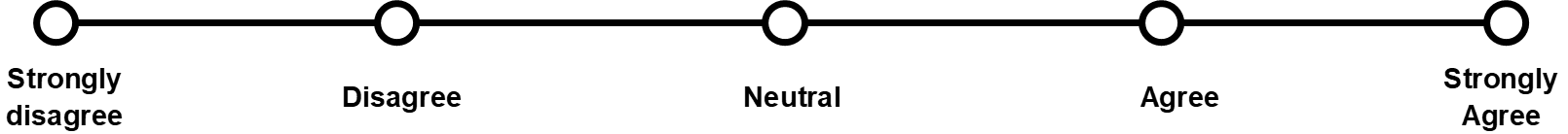


39. I do not see a problem with telepresenters contributing to telenursing (NEW)
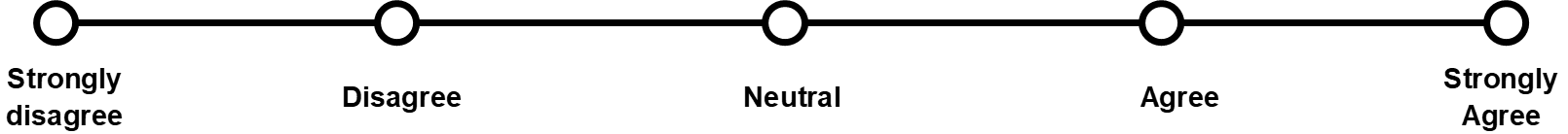


40. In general, I consider that the telenursing system easy to use (TAM3-PEOU3).


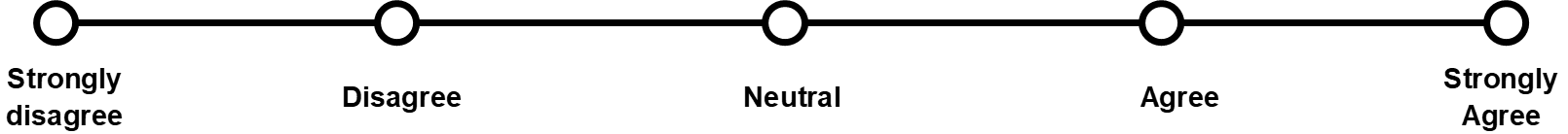


**Telecare procedures** (new)

Please indicate how much you agree to the following procedures being carried out via telenursing

41.Patient documentation for admission, transfer, and discharge procedures


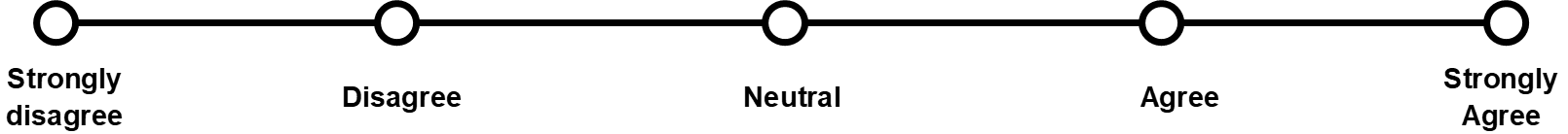


42.Monitoring, measurement, and evaluation of breathing


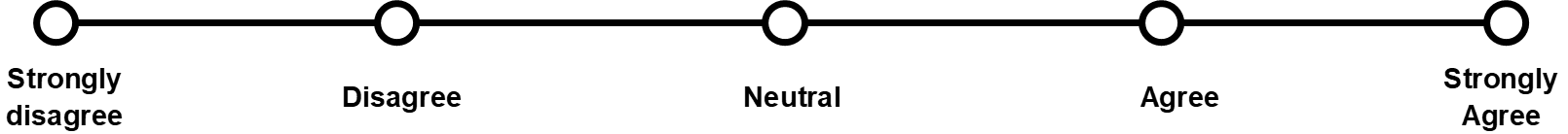


43.Monitoring, measurement, and evaluation of body temperature


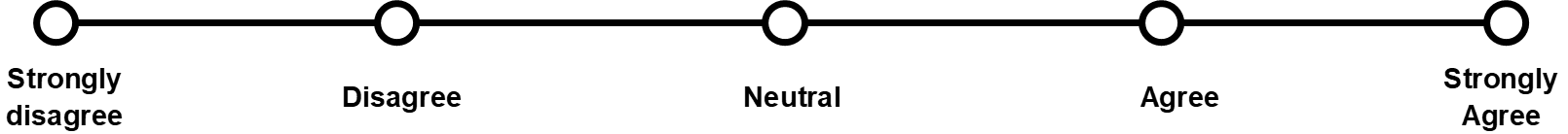


44.Monitoring, measurement, and evaluation of the heart rate


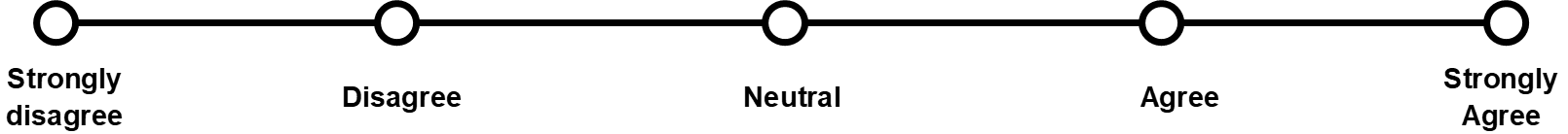


45.Monitoring, measurement, and evaluation of blood pressure


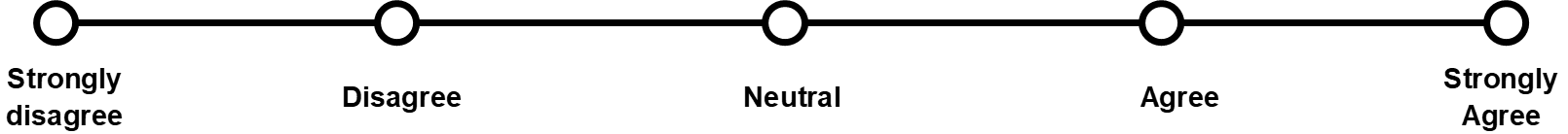


46.Monitoring, measurement, and evaluation of consciousness.


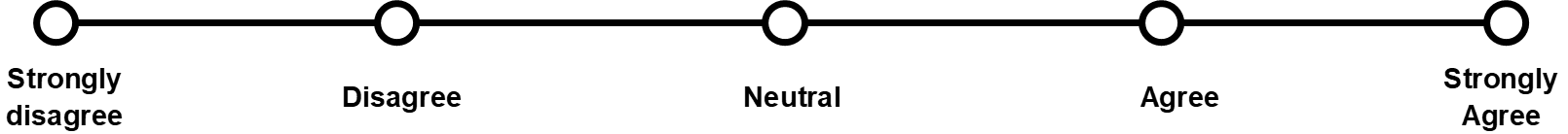


47.Monitoring, measurement, and evaluation of Central venous pressure.


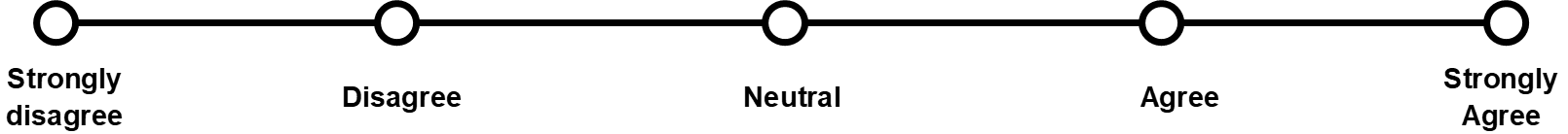


48. Record nursing history data.


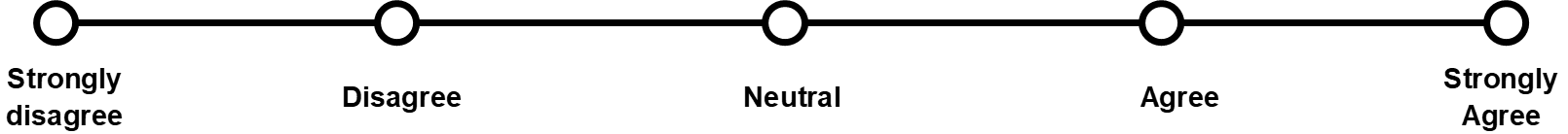


49.Visual patient surveillance (monitoring).


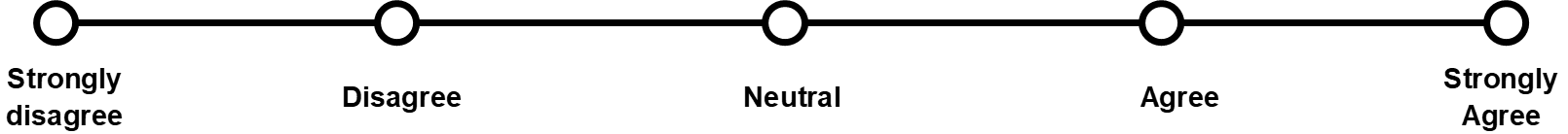


50.Tele control of IV fluid administration.


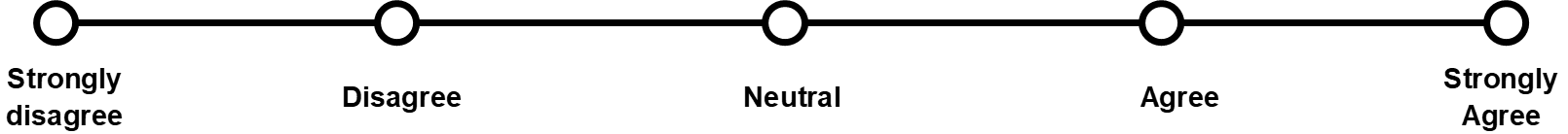


51.Patient control of medication administration.


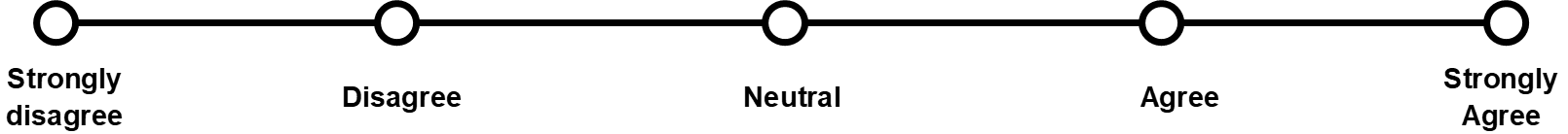


52. Storing control of medicines in nursing units.


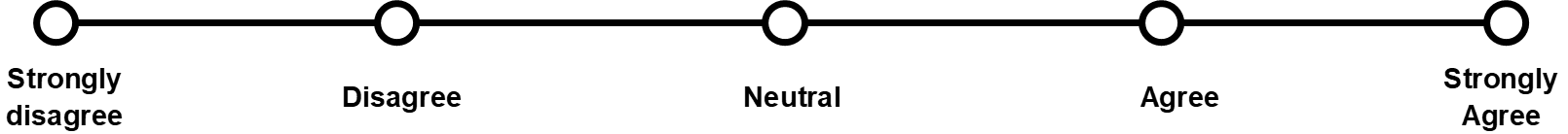


53.Patient education and advise.


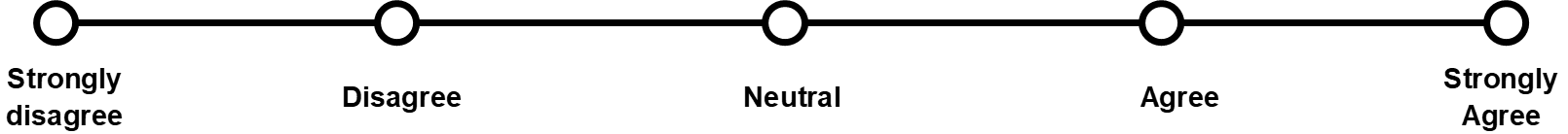


54.Psychological support.


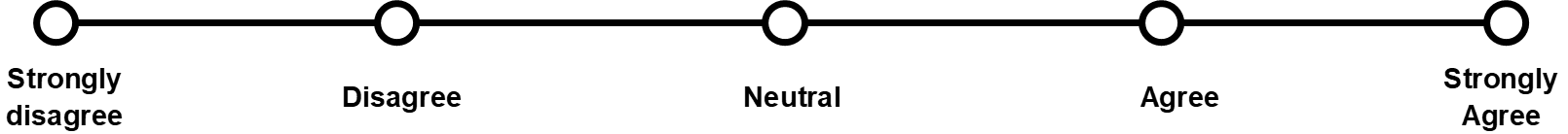


55. Diet control and education.


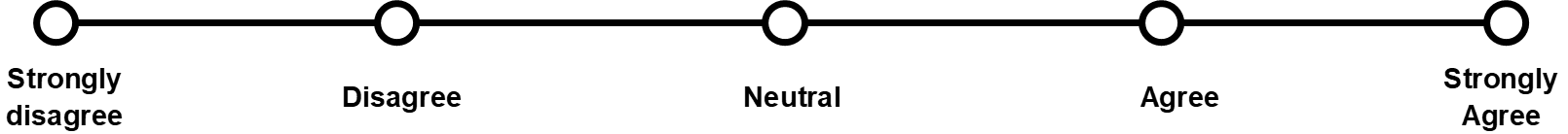


56. Oxygen administration control (monitoring)


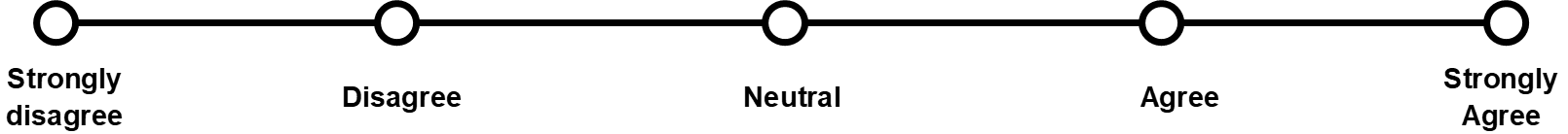


57. Urine control (Remote control device).


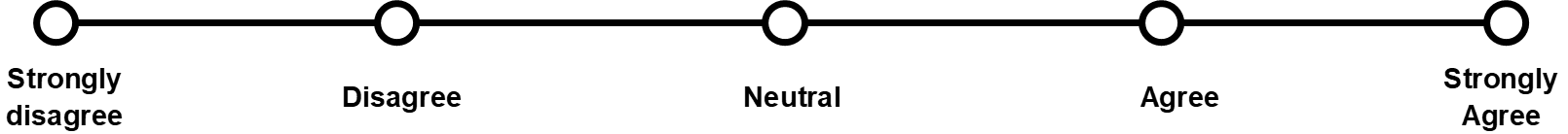


58. Pre- and post-operative communication with the patient.


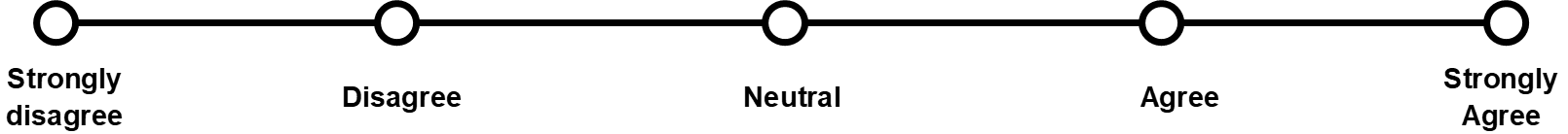


59. Developing or contributing to the development of unique-tailored patient care plans


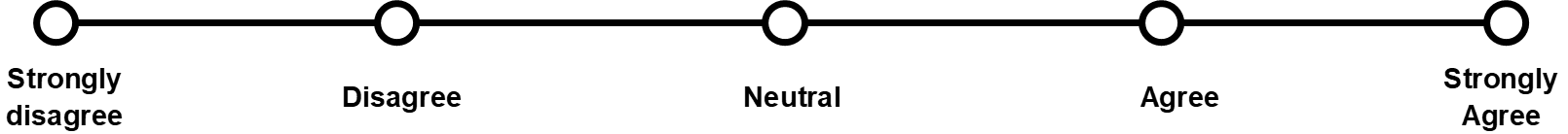


60. Educate patients and their families on how to follow their prescribed treatment and recovery instructions upon returning home


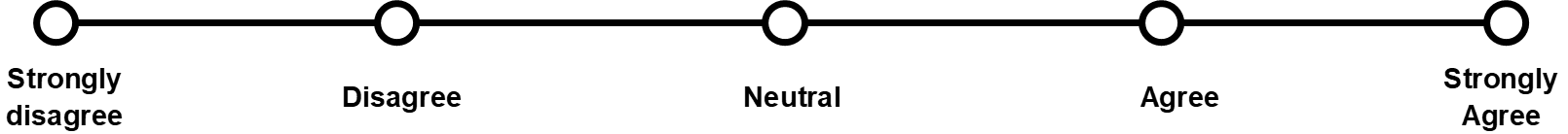

Supplement: Multimedia component 1 [file mmc1.docx]
